# Mouse *Tenm4* is required for mesoderm induction

**DOI:** 10.1186/1471-213X-13-9

**Published:** 2013-03-25

**Authors:** Hisashi Nakamura, Rita N Cook, Monica J Justice

**Affiliations:** 1 Department of Molecular and Human Genetics, Baylor College of Medicine, Houston, Texas 77030, USA

**Keywords:** Mouse, *Tenm4*, Gastrulation, Mesoderm induction, Anterior-posterior patterning, Bipolar disorder, Wnt signaling and ENU mutagenesis

## Abstract

**Background:**

*Tenm4* is a mouse homolog of the *Drosophila* gene Tenascin-m (Ten-m (*Odd oz*)), which functions in motor neuron routing. Recently, a genome-wide association analysis for bipolar disorder identified a new susceptibility locus at TENM4 increasing the importance of understanding *Tenm4*. A series of *Tenm4* mouse alleles showing a broad range of phenotypes were isolated after ENU mutagenesis. Here, we examine the timing and features of gastrulation failure in a loss of function allele.

**Results:**

Embryonic mesoderm did not form in loss of function *Tenm4*^*m1*/*m1*^ mutant embryos. Genes normally expressed in embryonic mesoderm were not expressed in the mutant, the primitive streak did not form, and markers of the anteroposterior axis were not expressed or were mislocalized. The lack of embryonic mesoderm could not be attributed to poor proliferation of the epiblast, as normal numbers of dividing cells were observed. Epiblast cells maintained expression of *Pou5f1* suggesting that they remain pluripotent, but they did not have the capacity to form any germ layer derivatives in teratomas, showing that the inability to induce mesoderm is cell autonomous. Misexpression of E-cadherin and N-cadherin suggest that the embryos did not undergo an epithelial-to-mesenchymal transition. In addition, Wnt signaling did not occur in the mutants, as assessed by the TOPGAL reporter assay, while a GSK3β inhibitor partially rescued the mutant embryos, and rescued TOPGAL reporter expression.

**Conclusions:**

These data demonstrate that *Tenm4* mutants fail to form a primitive streak and to induce embryonic mesoderm. Markers of anterior posterior patterning fail to be expressed or are mislocalized. Further, *Tenm4* mutants lack the ability to differentiate in a cell autonomous manner. Together, our data suggest that embryos become impaired prior to E6.5 and as a result, Wnt signaling fails to occur; however, the involvement of other signaling pathways remains to be examined.

## Background

Tightly controlled signaling pathways play critical roles in the early stages of embryogenesis and body plan formation. During gastrulation, morphogenetic movements in addition to cell proliferation and differentiation transform an embryo with a single germ layer into one with three layers, the ectoderm, mesoderm and endoderm. In the mouse, gastrulation initiates at E6.5, as cells of the primitive ectoderm (epiblast) delaminate at the posterior side of the embryo and ingress through the primitive streak in an epithelial-to-mesenchymal transition [1,2]. Genetic approaches have identified many genes that play a role in gastrulation and/or pattern formation [3]. The evolutionarily conserved signal transduction cascade of Wnt/β-catenin regulates patterning of the visceral endoderm, the induction of the primitive streak, and the formation of anterior neural ectoderm [4,5]. *Nodal* and *Brachyury* (T) are among the genes downstream of Wnt signaling that are required for primitive streak formation [5-8].

*Tenm4*, teneurin transmembrane protein 4, is a mouse homolog of the *Drosophila* gene tenascin major (*Ten-m)*, also called *Odd oz* (*Odz*) [9-11], which was originally associated with segmentation defects. Ten-m is a member of the Teneurin protein family and encodes a transmembrane protein with tenascin-like EGF (epidermal growth factor) repeats. Ten-m is found in both secreted and membrane-bound forms at several compartment boundaries, suggesting a role in cell-cell communication. Mutant *Ten-m Drosophila* embryos have abnormalities in neural ectoderm and mesoderm-derived tissues that result in death at the larval stage [9,10]. Recently, new alleles of *Drosophila Ten-m* were identified and characterized establishing that mutations in this gene do not cause segmentation defects; instead, *Ten-m* functions in motor neuron routing [12]. Ten-m is expressed in the central nervous system and epidermal stripes at stages when the growth cones of intersegmental neurons (ISNs) navigate to their targets. Both mutation and over-expression of Ten-m in epidermal cells leads to ISN misrouting [12]. A related protein, tenascin a (Ten-a) has a trans-synaptic signaling role with Ten-m: Ten-a is presynaptic whereas Ten-m is predominantly postsynaptic in neuromuscular synapse organization and target selection [13].

The mouse has four teneurin transmembrane protein family members (*Tenm1-4*) that lack signal peptides at the N-terminus, but contain a short hydrophobic domain characteristic of transmembrane proteins followed by a region with eight EGF-like repeats, and a large C-terminal domain [14,15]. The vertebrate homolog of *DrosophilaTen-a* is called *Tenm1* and the homolog of *Drosophila Ten-m* is *Tenm4*. All four mammalian *Tenm* genes are highly expressed in the brain and each gene produces many alternatively spliced transcripts, suggesting a variety of protein functions in different tissues [16,17]. Novel mutant alleles having defects in early mouse embryonic development can be identified using ethylnitrosourea (ENU) mutagenesis [18]. A series of ENU-induced alleles at mouse *17Rn3*[19-21] contain mutations in the *Tenm4* gene [16], which exhibit a wide array of phenotypes, ranging from embryonic death at gastrulation to viable with skeletal defects [16]. In two loss-of-function alleles (*Tenm4*^*m1*^ and *Tenm4*^*m2*^), embryos fail to gastrulate. In less severe alleles *(Tenm4*^*m3*^*, Tenm4*^*m4*^ and *Tenm4*^*m5*^), gastrulation occurs, but body axis formation, somitogenesis, vasculogenesis, cardiogenesis, and fusion of the allantois with the chorion are disrupted leading to death at early- to mid-gestation stages. Consistent with these mutant phenotypes, *Tenm4* is ubiquitously expressed in the epiblast and extraembryonic regions as early as E6.5. By E7.5, *Tenm4* is highly expressed in the mesoderm of the developing embryo and extraembryonic tissues. Later, *Tenm4* is expressed mainly in the neuroectoderm, but expression is maintained in the tail bud, somites and limbs [16].

To begin to address the biological function of *Tenm4*, the loss of function allele *Tenm4*^*m1*^, and a hypomorphic allele *Tenm4*^*m4*^ were examined. *Tenm4*^*m1/m1*^ mutant embryos failed to initiate gastrulation, failed to form a primitive streak, and failed to develop mesoderm. In addition, *Tenm4*^*m1/m1*^ mutant embryos were incapable of forming any differentiated tissue. An analysis of the hypomorphic allele, *Tenm4*^*m4*^, along with the null allele, determined that mutant cells did not properly express E- or N-cadherin, suggesting that the epithelial-to-mesenchymal transition (EMT) did not occur. Moreover, the mutants failed to up-regulate a TOPGAL reporter gene, suggesting that Wnt signaling failed to occur. Finally, a GSK3β inhibitor rescued the ability of the embryos to form extraembryonic mesoderm and partially rescued their ability to form embryonic mesoderm.

## Results

### Tenm4^m1/m1^ mutant embryos arrest at the gastrulation stage

**Figure 1:**
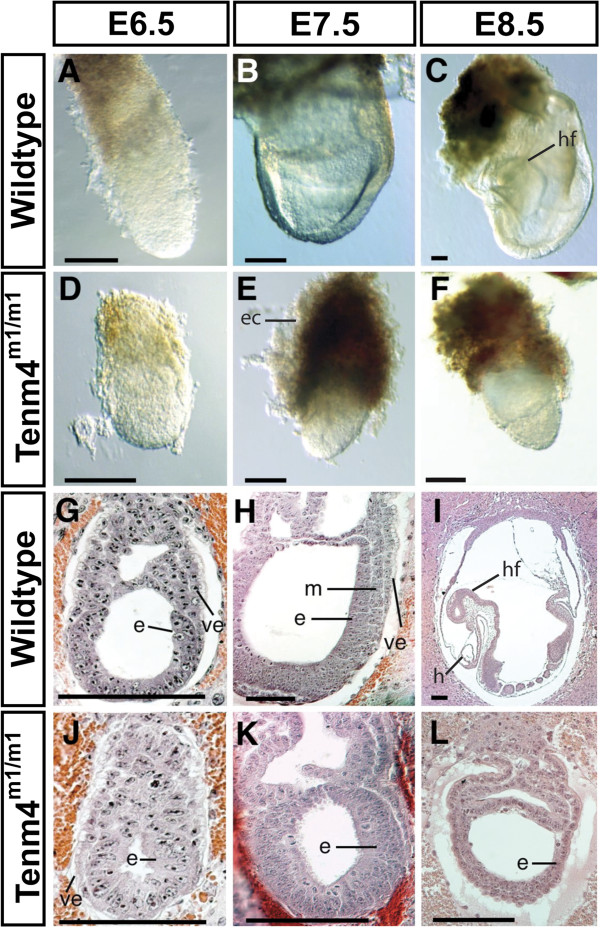
***Tenm4***^***m1/m1 ***^**mutant embryos arrest at the gastrulation stage and fail to develop a mesoderm.** Wildtype and *Tenm4*^*m1/m1*^ mutant embryos at E6.5 – 8.5 (**A**-**F**). *Tenm4*^*m1/m1 *^mutant embryos (**D**) are similar to wildtype littermates at E6.5 (**A**). E7.5 *Tenm4*^*m1/m1 *^mutant embryos (**E**) have a poorly defined extraembryonic region. E8.5 wild-type embryos (**C**) start organogenesis, whereas the *Tenm4*^*m1/m1 *^mutant embryos (**F**) do not develop or grow. Histological paraffin sections were prepared from wildtype littermates (**G-I**) and *Tenm4*^*m1/m1 *^mutant embryos (**J-L**), and stained with hematoxylin and eosin. Sagittal sections of heterozygote and *Tenm4*^*m1/m1 *^embryos at days E6.5 (**G** and **J**), E7.5 (**H** and **K**) and E8.5 (**I** and **L**). Thickness of all sections is 7 mm. Epiblast (e), ectoplacental cone (ec), heart (h), head fold (hf), mesoderm (m), and visceral endoderm (ve). Bar 100 μm. PCR genotyping was used to identify the mutant classes. Developmental stages and genotypes are indicated at the top and left of the panels, respectively.

At E6.5, wildtype and *Tenm4*^*m1/m1*^ embryos were similar in appearance (Figure 1A and D). At E7.5, mutant embryos were approximately half the size of littermates (Figure 1B and E), and at E8.5, mutant embryos were arrested in development at the E6.5 stage (Figure 1C and F). Prior studies showed that most embryos were dead or dying by E8.5 and that no mutant embryos survived past E9.5 [16]. Histology revealed phenotypic differences between wildtype and mutant embryos (Figure 1G-L). At E6.5 wild-type embryos had a well-organized ectoderm, visceral endoderm, and extraembryonic mesoderm with proamniotic and extraembryonic cavities (Figure 1G). In contrast, mutant embryos had no sign of mesoderm (Figure 1J). At E7.5, wildtype embryos developed embryonic mesoderm (Figure 1H). By E8.5, wildtype embryos developed three primitive embryonic cavities, including the amniotic, exocoelomic and ectoplacental cavities, head folds, and embryonic and extraembryonic mesodermal tissues, including the allantois (Figure 1I). However, no mesoderm formed and the embryonic region did not expand in *Tenm4*^*m1/m1*^ mutant embryos by E7.5 or 8.5, although some embryos appeared to form a morphologically abnormal extraembryonic cavity, perhaps caused by the expansion of the extraembryonic ectoderm and visceral endoderm (Figure 1K and L).

### Tenm4^m1/m1^ mutants do not produce mesoderm

**Figure 2:**
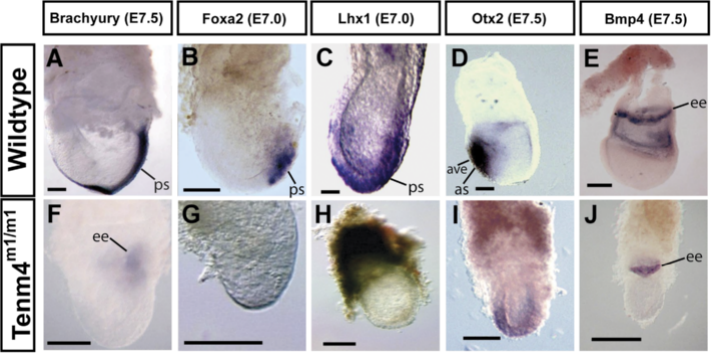
**Mesodermal and anterior marker gene expression in *Tenm4***^***m1/m1 ***^**mutant embryos. **Whole-mount *in situ *hybridization was performed to examine the expression of marker genes (*Brachyury*, *Foxa2*, *Lhx1*, *Otx2 and Bmp4*). Wildtype embryos are oriented with the anterior side of embryos on the left. A-P axis orientation may vary because the A-P axis was not specified in mutants. *Brachyury *is expressed in the primitive streak and notochord of an E7.5 wildtype embryo (**A**). *Tenm4*^*m1/m1 *^mutants at E7.5 lack *Brachyury *expression in the posterior embryonic region (**F**), although a weak signal is detected in extraembryonic tissue. *Foxa2* is expressed in the primitive streak in an E7.0 wildtype embryo (**B**), but not in *Tenm4*^*m1/m1 *^(**G**). *Lhx1 *is expressed in mesoderm wings and anterior visceral endoderm at E7.0 (**C**), but not in *Tenm4*^*m1/m1 *^(**H**). *Otx2 *is restricted to the anterior epiblast of an E7.5 wildtype embryo (**D**), but not restricted in *Tenm4*^*m1/m1 *^(**I**). *Bmp4 *is expressed in extraembryonic ectoderm and extraembryonic mesoderm at E7.5 (**E**). *Tenm4*^*m1/m1 *^mutant shows ectodermal expression but not mesodermal (**J**). Anterior streak (as), anterior visceral endoderm (ave), extraembryonic ectoderm (ee), primitive streak (ps). Bar 100 μm.

*Brachyury* is expressed prior to the onset of gastrulation at E6.5 in the extraembryonic ectoderm adjacent to the epiblast, and shifts to the developing primitive streak, where it is a marker of mesodermal-derived notochord [22,23]. No *Brachyury* expression was found in the embryo of *Tenm4*^*m1/m1*^ mutants, although a weak signal was observed in the extraembryonic tissue in half of the mutants (Figure 2A, F and Additional file 1: Figure S1). The cause of the inconsistent extraembryonic expression is not clear. This is an ENU-induced point mutation, and often such alleles are somewhat leaky due to read-through of the point mutation. Alternatively, this observation is consistent with expression in extraembryonic ectoderm, indicating developmental delay. Importantly, none of the mutants had *Brachyury* expression in the area of the potential primitive streak. Additional primitive streak markers were examined. In wildtype embryos, *Foxa2* (*Hnf3β)* expression is restricted to the anterior end of the primitive streak at E7.0 (Figure 2B and [24-26]. *Lhx1* is expressed at low levels in cells entering the streak and at high levels in cells exiting the streak, and similarly, in anterior endoderm at E7.5 (Figure 2C and [26,27]. In *Tenm4*^*m1/m1*^ mutants, neither *Foxa2* nor *Lhx1* expression was detected (Figure 2G and H, respectively).

To determine how the *Tenm4*^*m1*^ mutation affects the development of the anterior patterning of the embryo, the expression pattern of *Otx2* was examined, which is expressed uniformly in the epiblast and the anterior visceral endoderm (AVE) prior to gastrulation. The expression of *Otx2* becomes restricted to the anterior epiblast as mesoderm migrates from the primitive streak suppressing its expression in the posterior epiblast (Figure 2D and [28]). In *Tenm4*^*m1/m1*^ embryos, *Otx2* expression does not become fully restricted to the anterior epiblast, consistent with the lack of mesoderm influencing its restricted expression (Figure 2I). *Bmp4* is expressed in the extraembryonic ectoderm adjacent to the proximal epiblast prior to gastrulation and later in the posterior primitive streak and extraembryonic mesoderm (Figure 2E and [29]. In *Tenm4*^*m1/m1*^ mutants, the expression of *Bmp4* is limited to a proximal ring at the junction of the embryonic and extraembryonic ectoderm, confirming the absence of posterior mesoderm (Figure 2J).

### Proliferation and apoptosis of epiblast cells in Tenm4^m1/m1^ is similar to wild type

**Figure 3:**
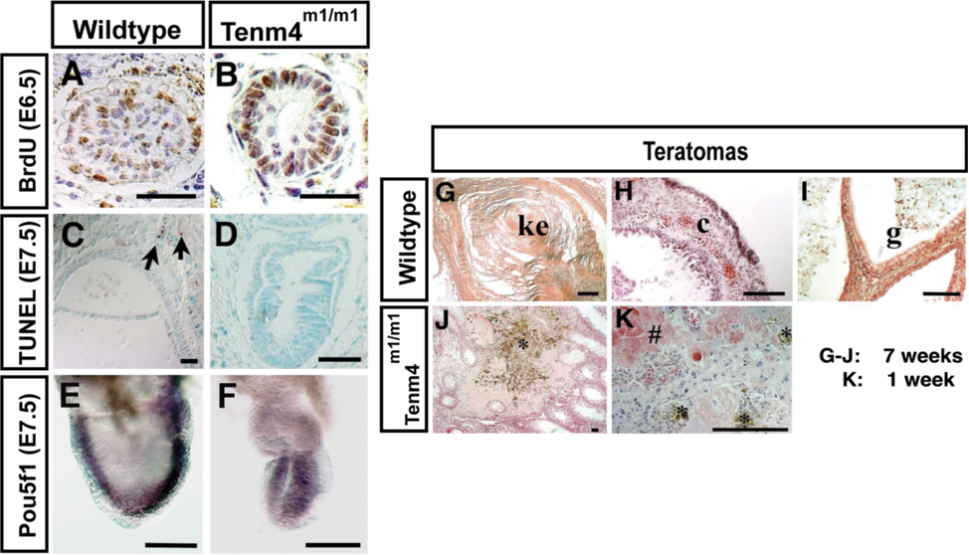
***Tenm4***^***m1 ***^**cells lack the ability to differentiate. **Cell proliferation of the epiblast is affected during gastrulation as evidenced by immunostaining of BrdU in wildtype (**A**) and *Tenm4*^*m1/m1 *^(**B**) embryos at E6.5. Since sections were counterstained with hematoxylin, BrdU-negative nuclei are blue. TUNEL assay of normal (**C**) and *Tenm4*^*m1 *^(**D**) embryos at E7.5. Whole-mount immunostaining using an anti-Pou5f1 antibody in wildtype (**E**) and *Tenm4*^*m1/m1 *^(**F**) embryos at E7.5. Teratomas derived from wildtype and *Tenm4*^*m1/m1 *^embryos fail to differentiate in a cell autonomous manner. Histological sections of 7 week old teratoma derived from wildtype embryo (**G-I**), 7 week old teratoma derived from *Tenm4*^*m1/m1 *^(**J**) and 1 week old teratoma derived from *Tenm4*^*m1/m1 *^(**K**). Keratinized epithelium (ke) in wildtype teratoma (**G**). Undefined tissue, which may be cartilage (c) in wildtype teratoma (**H**). Endodermal tissue, which may be gut (g) in wildtype teratoma (**I**). Degenerated teratoma derived from *Tenm4*^*m1/m1 *^(**J** and **K**). Inflammation with dead blood cells (*) indicating degradation of teratoma. Blood cells invading into teratoma (#). Bar 50 μm (**A**-**D**), 100μm (**E**-**K**).

A threshold number of epiblast cells must be attained and maintained for gastrulation to initiate and progress [3]. Mouse embryos that lack a normal number of cells due to abnormal cell proliferation or to cell losses will delay gastrulation until the embryo attains the appropriate number of cells. Mutant embryos that fail to sustain the proliferation of epiblast cells do not initiate gastrulation or will arrest after the formation of a rudimentary primitive streak. To examine the effect of the *Tenm4*^*m****1***^ mutation on cellular proliferation *in vivo*, the incorporation of 5^′^-bromo-2^′^-deoxyuridine (BrdU) into DNA was analyzed. At E6.5 (Figure 3A, B and Table 1), 48% of the nuclei of wildtype or heterozygous epiblast cells were labeled with BrdU after a 20-minute exposure. In *Tenm4*^*m1/m1*^ mutant embryos, 47% of the nuclei were labeled, suggesting that the proliferation of epiblast cells is normal at this time point. These data suggest that the gastrulation defect is not caused by insufficient cell proliferation in the epiblast. However, the total number of mutant epiblast cells was lower at E6.5 (Figure 3B and Table 1), and a student’s t-test indicates that total cell numbers are significantly different (t = 0.002). This suggests that the growth of the embryo starts to become impaired prior to E6.5. Increased apoptosis could also cause a reduced number of cells, so the TUNEL assay was used to examine cell death. Normally, cell death occurs primarily in extraembryonic tissues, especially in trophoblast (Figure 3C), but is rare in embryonic cells. Very few apoptotic cells were observed in mutant embryos at E6.5 (Data not shown) or at E7.5 (Figure 3D and Table 1).

**Table 1:** ***Tenm4***^***m1/m1 ***^**mutant embryo BrdU incorporation and TUNEL assay results**


**i) BrdU (E6.5)**	**wt or m1/+ (n=24)**	**m1/m1 (n=13)**
Total cells (per section)	88.2 (±11.5)	62.2 (±4.66)
BrdU labeled cells	42.1 (±6.41)	29.2 (±3.67)
Labeling index (%)	47.7 (±4.45)	46.9 (±2.59)
**ii) TUNEL assay (E7.5)**	**wt or m1/+ (n=10)**	**m1/m1 (n=6)**
Embryonic region	1.2 (±0.44)	0.17 (±0.41)
Extraembryonic region	11.3 (±3.11)	0.50 (±0.84)

i) BrdU incorporation at E6.5. ii) TUNEL assay at E7.5. Numbers in parentheses are standard deviation (±SD).

The epiblast contains a highly regulated stem cell population that adjusts to various perturbations including major alterations in cell number and cell position. Because the cells remain in an undifferentiated state until late gastrulation [30,31], one possibility for the low cell count in *Tenm4*^*m1/m1*^ at the late gastrulation stage is a precocious differentiation of epiblast stem cells. Therefore, the expression of *Pou5f1* at E7.5 was examined. *Pou5f1* is distinguished by exclusive expression in blastomeres, pluripotent cells, and the germ cell lineage [32-36]. At E7.5, *Pou5f1* expression should be restricted to the epiblast (Figure 3E). In mutant embryos, the expression of *Pou5f1* is similar to wildtype, indicating that undifferentiated pluripotent epiblast cells are present (Figure 3F).

To examine differentiation potential, wildtype and mutant embryonic tissues at E6.5 were transplanted into the testes of adult male mice and examined for the development of teratomas. After 7 weeks, the testes were fixed, sectioned and processed for histological analysis (Figure 3G-K). Teratomas derived from heterozygotes differentiated into many kinds of tissues, which are produced from all three germ layers (Figure 3G-I). However, the teratomas derived from *Tenm4*^*m1/m1*^ mutant embryos produced no differentiated tissues (Figure 3J). The size of teratomas derived from mutant embryos never exceeded 2 mm, whereas teratomas from wildtype embryos grew to 0.5-2 cm in diameter (Table 2). Furthermore, approximately half of the mutant-derived teratoma cells had degenerated when examined at 7 weeks. Only four homozygous mutant-derived teratomas survived to be genotyped out of 43 teratomas examined (9.3%), all of which had inflammatory reactions, indicating the decay of the cells. Moreover, six teratomas could not be genotyped because they were severely decomposed. Mutant-derived teratomas had signs of inflammation when teratomas were assessed at 1 week after transplantation, although again, no cell differentiation was observed (Figure 3K). Together, these results suggest that the failure to differentiate is cell autonomous. To further test this question, embryos were cultured to attempt to generate embryonic stem (ES) cells for studies of chimeras with mutant cells. Although multiple ES cell lines were derived from wildtype (4/55) or heterozygous (12/55) embryos, none were derived from mutants (0/55), precluding experiments on such cells. Blastocysts from matings of *Ten-m4*^*m1*^ heterozygotes were collected at E3.5 and cultured *in vitro* to observe any defects in the inner cell mass (ICM) and/or trophectoderm outgrowth. All blastocysts attached normally to gelatin-coated dishes and completely hatched from the zona pellucida after 2–3 days in culture. After hatching, the growth of the ICM and the extent of trophoblast outgrowth was very abnormal in *Ten-m4*^*m1/m1*^ mutants (Additional file 2: Figure S2). Mutant cells could be easily distinguished from those from wild type or heterozygotes because they failed to adhere and could not survive more than 8 days in culture.

**Table 2:** **Teratomas (7 weeks old and 1 week old) derived from *Tenm4***^***m1 ***^**mutant embryos**


**i) 7 weeks old**				
		+/+	m1/+	m1/m1
No. of transplantation		9	24	10
Tumor developed		9	24	4
Dead or degrading		0	0	6*
Tumor size				
large (1.0 - 2.0 cm)		8	24	0
middle (0.5 - 1.0 cm)		1	0	0
small (< 0.5 cm)		0	0	4
Differentiation				
Ectoderm		9	24	4
Mesoderm		8	21	0
Endoderm		4	14	2
**ii) 1 week old**				
		+/+	m1/+	m1/m1
No. of transplantation		3	8	5
Tumor developed		3	7	5
Dead or degrading		0	1	0
Tumor size				
large (1.0 - 2.0 cm)		0	0	0
middle (0.5 - 1.0 cm)		2	5	0
small (< 0.5 cm)		1	2	5
Differentiation				
Ectoderm		3	7	0
Mesoderm		2	5	0
Endoderm		2	4	0

Teratomas were assessed by histology after measuring the diameter of the teratoma. The teratoma sizes were categorized as large (1–2 cm), middle (0.5-1 cm) and small (less than 0.5 cm) and the tissue types in teratomas derived from normal and mutant embryos were categorized as ectoderm, endoderm and mesoderm. *Not genotyped due to degrading of teratoma tissue.

### Expression of E-cadherin, N-cadherin and Snai1

**Figure 4:**
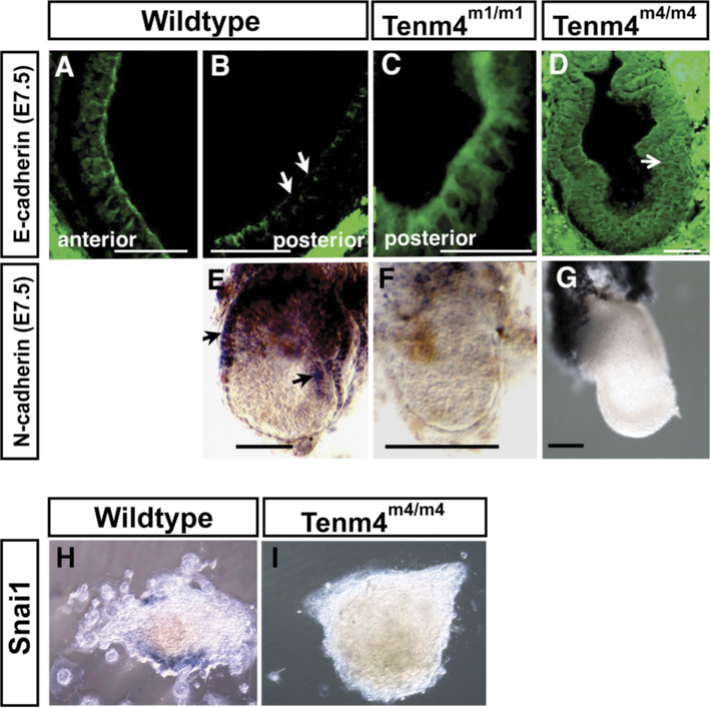
**E-cadherin, N-cadherin and Snai1 expression.** E-cadherin is down-regulated in the primitive streak and mesoderm in wildtype embryos (**B**, white arrows), but not in *Tenm4*^*m1/m1*^ (**C**) or *Tenm4*^*m4/m4*^ (**D**). Note the formation of mesoderm in the m4 allele (white arrow), while E-cadherin fails to be down-regulated. N-cadherin is expressed in the visceral endoderm and the primitive streak in wildtype embryos (**E**); however, neither *Tenm4*^*m1/m1*^ nor *Tenm4*^*m4/m4*^ mutants show expression of N-cadherin (**F** and **G**). *Snai1* is expressed in ectoplacental cone explants cultured from E6.5 wildtype (**H**) but not in those from *Tenm4*^*m4/m4*^ (**I**) embryos. Bar 50 μm (**A-D**), 100μm (**E-G**).

An epithelial-to-mesenchymal transition (EMT) is an essential step in gastrulation, which requires Wnt signaling to proceed. Down-regulation of E-cadherin is an early step of the EMT. Embryos that fail to gastrulate do not undergo the EMT; however, the *Tenm4* allelic series includes a hypomorphic allele, *Tenm4*^*m4/m4*^, which gastrulates to form mesodermal tissues, including yolk sac, heart and blood vessels, yet arrests in development by E8.5 [16]. The m4 allele allows the EMT to be assessed in a mutation that does not arrest before gastrulation occurs. The expression of E-cadherin in the loss of function allele *Tenm4*^*m1/m1*^ and the hypomorphic allele *Tenm4*^*m4/m4*^ was examined. E-cadherin was absent in the primitive streak and mesoderm of wildtype embryos, (Figure 4B), whereas E-cadherin expression was not down regulated in the epiblast of both *Tenm4*^*m1/m1*^ and *Tenm4*^*m4/m4*^ (Figure 4C and D, respectively). When epiblast cells delaminate at the primitive streak to form mesoderm, they switch expression from E- to N- cadherin. N-cadherin was expressed at high levels in the yolk sac and primitive streak in wildtype embryos (Figure 4E); however, no N-cadherin expression was observed in either *Tenm4*^*m1/m1*^ or *Tenm4*^*m4/m4*^ (Figure 4F and G). *Snai1* (mSnail) suppresses E-cadherin to initiate migration of trophoblast giant cells, which also requires the EMT. No *Snai1* expression was observed in cultured ectoplacental cone mutant explants (Figure 4H and I). All wildtype explants expressed *Snai1* (100%; n=15), whereas only one *Tenm4*^*m1/*m1^ explant had low *Snai1* expression, and five had none (n=6), 16.7%.

### The TOPGAL assay suggests that Wnt signaling does not occur

**Figure 5:**
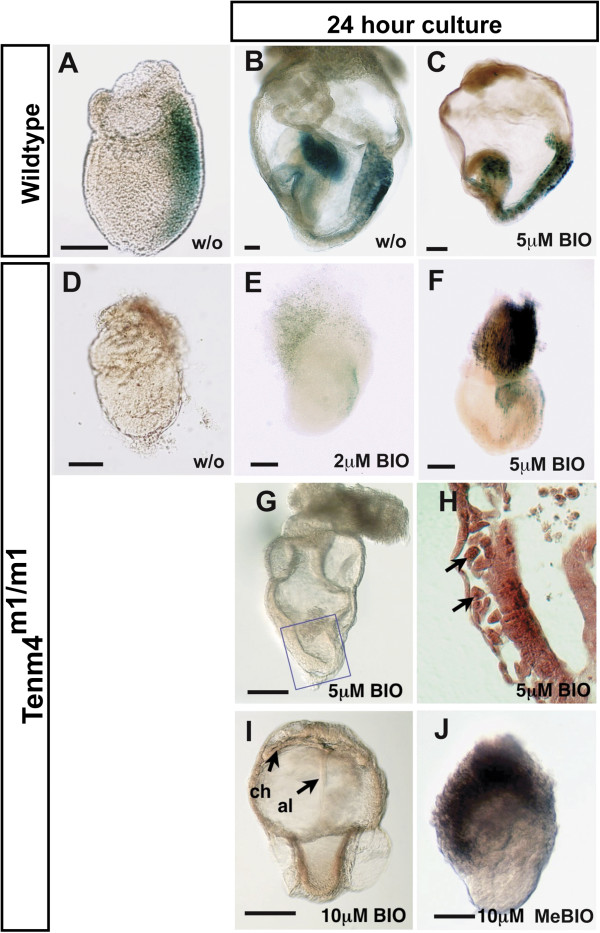
**Partial rescue of TOPGAL reporter expression by GSK inhibitors in *Tenm4***^***m1/m1***^**mutants.** Shown are E7.5 embryos removed from the maternal environment, then cultured for 24 hours without addition of BIO or MeBIO (w/o), with 2, 5 or 10 μM 6-bromoindirubin-3^′^-oxime (BIO), or 10 μM 1-methyl-6-bromoindirubin-3^′^-oxime (MeBIO). The TOPGAL reporter transgene was used to assess WNT signaling by staining for β-galactosidase activity. Wildtype embryos obtained at E7.5 (**A**) and cultured for 24 hours (**B** and **C**) had β-galactosidase expression without or with the addition of BIO. *Tenm4*^*m1/m1*^ mutant embryos had no β-galactosidase expression when obtained at E7.5 (**D**) but expressed β-galactosidase after 24 hours in 2 μM (**E**) or 5 μM BIO (**F**). A *Tenm4*^*m1/m1*^ mutant embryo cultured 24 hours in 5 μM BIO, not stained for β-galactosidase to show morphological features is shown in **G** alongside a histological section (**H**) from the area in the blue box showing migrating mesodermal cells (arrowheads). A *Tenm4*^*m1/m1*^ mutant embryo cultured 24 hours with 10 μM BIO shows fusion of the chorion (ch) with the allantois (al) (**I**), also not stained with β-galactosidase. No embryos cultured with MeBIO showed evidence of mesoderm or TOPGAL activity, a representative one cultured in 10uM MeBIO is shown in (**J**). Bar 100 μm.

*Drosophila Ten-m* affects wingless expression [9]. In mouse, canonical signaling by Wnt3 is required for mesoderm induction in the early embryo [5]. A TOPGAL transgenic expression reporter mouse strain, which expresses β-galactosidase under the control of three copies of the Wnt-specific LEF/TCF binding sites was used to assess Wnt signaling in the mutants [37]. In wild-type E7.5 embryos, TOPGAL activity was detected in the primitive streak (Figure 5A and B). In contrast, TOPGAL activity was not present in *Tenm4*^*m1/m1*^ E7.5 embryos (Figure 5D).

Normally, glycogen synthase kinase 3 (GSK3) is inhibited by Wnt signaling to promote stabilization of β-catenin and transcription of target genes [38]. To attempt to rescue the defective mesoderm induction in *Tenm4*^*m1/m1*^ mutant embryos, Wnt signaling was ectopically induced with a GSK3 specific inhibitor, 6-bromoindirubin-3^′^-oxime (BIO). Its analog, 1-methyl-6-bromoindirubin-3^′^-oxime (MeBIO) is a useful negative control [39]. When MeBIO was added to the embryo culture medium, *Tenm4*^*m1/m1*^ mutant embryos did not produce any mesodermal cells (Figure 5J). However, when BIO was added at 2 μM and 5 μM, extraembryonic tissue grew (Figure 5E, F and G). Even so, the embryonic region did not expand, although some cells migrated away from the epiblast (Figure 5G and H). Using a higher BIO concentration (10 μM), *Tenm4*^*m1/m1*^ mutant embryos produced allantois and chorion, which fused, suggesting that extraembryonic mesoderm was produced and differentiated (Figure 5I). Moreover, TOPGAL signaling was induced to various degrees in both embryonic and extraembryonic tissues (Figure 5E, F, G and I), suggesting that Wnt signaling was restored in some cells.

## Discussion

Here we show using mouse mutants that the earliest functions of *Tenm4* are prior to gastrulation such that Wnt signaling does not occur. A loss of function allele failed to gastrulate and produced no mesoderm. *In vivo* and *in vitro* experiments showed that loss of function mutant embryos did not have the potential to form differentiated tissues, a defect that was cell autonomous. Further, E-cadherin and N-cadherin expression was abnormal in both loss of function and hypomorphic alleles, supporting the idea that *Tenm4* mutant cells fail to undergo the epithelial-to-mesenchymal transition, surprisingly even when a primitive streak forms and gastrulation occurs. The formation of embryonic cavities, along with weak expression of *Brachyury* in extraembryonic regions and rescue of extraembryonic mesoderm by GSK inhibitors, suggests that extraembryonic mesoderm may remain competent in the mutants; however, mesoderm in the embryo proper may lack the potential to differentiate.

The phenotype of *Tenm4*^*m1/m1*^ mutant embryos is distinct from other mice with gastrulation failure. Mutations in *Nodal*, *Smad2*, *Smad4* or *Actr1b*, which block signaling of members of the transforming growth factor beta (TGF-β) family, affect embryonic differentiation at the egg cylinder stage before gastrulation [6,40-43]. Smad2-dependent Nodal/activin/TGF-β signaling is essential for the maintenance of pluripotency in the epiblast and in human embryonic stem cells [44]. *Tenm4*^*m1*^ deficient embryos maintain expression of *Pou5f1* in the epiblast, suggesting that the epiblast has pluripotent potential. *Foxh1* and *Cripto* mediate the Nodal-signaling pathway [45,46]. In contrast to *Tenm4*^*m1*^, *Foxh1*- and *Cripto*-deficient embryos produce a primitive streak. Bone morphogenic protein (BMP) signaling is also required for gastrulation, and mesoderm induction fails in type-I BMP receptor (*Bmpr1*) mutants [47]. In contrast to *Tenm4* mutants, teratomas derived from *Bmpr1*^-/-^ embryos produce mesoderm-derived tissues [47]. BMP signaling is required for visceral endoderm differentiation and the formation of cavities in the early mouse embryo [48]. *Tenm4*^*m1/m1*^ embryos developed normal visceral endoderm and embryonic cavities, as they maintained *Bmp4* expression in the extraembryonic ectoderm. Taken together, these data suggest that the first embryonic function of *Tenm4* may not be to target TGF-β or BMP signaling.

Canonical Wnt signaling is essential for mesoderm formation, embryonic patterning, and epithelial-to-mesenchymal interactions. *Wnt3* plays a role in inducing mesoderm and forming the primitive streak [49,50]. Although *Wnt3* mutants fail to induce mesoderm, the visceral endoderm and epiblast layers continue to grow and expand [5]. In contrast, *Tenm4*^*m1/m1*^ embryos failed to expand the visceral endoderm and epiblast layers. *Mesd*, which encodes the chaperone for the Wnt co-receptors LRP5/6, as well as a double knockout of LRP5/6, shows phenotypes similar to the *Tenm4*^*m1*^ mutant [49,50]. In these mutants, defects in extraembryonic tissues lead to failure to organize the proximal epiblast, similar to *Tenm4*; however, in contrast, the visceral endoderm and epiblast layers continue to grow, similar to *Wnt3* mutants. β-catenin regulates *Cripto*- and *Wnt3*-dependent gene expression programs in mouse anterior posterior axis and mesoderm formation, controlling both the Wnt and nodal pathways [51-53]. No mesoderm or head structures formed in β-catenin-deficient embryos, and markers of posterior mesoderm differentiation such as *Brachyury*, as well as markers of A-P axis formation such as *Hex* and *Hesx1*, were not expressed [54]. Similar to β-catenin- and *Wnt3*-deficient embryos, *Tenm4*^*m1/m1*^ mutants failed to form a primitive streak, even though the epiblast maintained high *Pou5f1* expression, unlike mutations in *Smad2*, *Smad4* and *Nodal*. The *Tenm4*^*m1/m1*^ mutant phenotype is more similar to canonical Wnt mutants than those of other pathways, yet seems to lack competency for the embryonic epiblast to differentiate into mesoderm [55].

*Fgfr1* mutants that do not activate *Snai1* show defective mesoderm migration and differentiation [56]. The suppressor of E-cadherin, *Snai1*, was not up-regulated in cultured mutant ectoplacental cone explants of the hypomorphic mutant *Tenm4*^*m4*^. These data, combined with knowledge of *Tenm* family EGF structural motifs, membrane orientation, and known secreted and membrane-bound forms, suggest that *Tenm4* may be an important molecule for proper adhesion of migrating extraembryonic cells. Embryo cultures indicated that a GSK3β inhibitor induced the outgrowth of extraembryonic tissues, and partially rescued defective mesoderm induction, particularly in extraembryonic tissues. The activity of GSK inhibitors is not restricted to Wnt signaling, and in fact, can influence cell adhesion molecules [57]. Furthermore, the inhibitors did not fully rescue the mutant phenotypes, suggesting that other pathways are likely to be involved.

The Wnt pathway has evolved to interact with many receptors in a variety of signaling pathways that are independent of canonical signaling, see [58] for review. For example, non-canonical Wnt signaling is required for planar cell polarity and convergent extension [58]. In addition to its essential roles in gastrulation, stem cell homeostasis and axis patterning, the Wnt pathway plays a key role in neuronal development and maintenance. In particular, non-canonical Wnt signaling is crucial for path finding during axonal navigation as well as for synapse formation [58,59]. Drosophila Ten-m binds with filamins via its filamin-binding domain to reorganize the cytoskeleton during axon guidance [12], a function of non-canonical Wnt signaling. Ten-m is expressed in muscle, but interacts with Ten-a at the neuromuscular synapse, evidencing its role in cell-cell communication during synapse organization and function [13]. Recently, a large-scale genome-wide association study (GWAS) identified an intronic variant in TENM4 (ODZ4) as being associated with bipolar disorder, a severe mood disorder that affects over 1% of the population [60]. Wnt signaling is implicated in many neuropsychiatric disorders, including schizophrenia, bipolar disorder and autism [59]*.* Therefore, it will be intriguing to determine if *Tenm4* has a direct role in Wnt signaling; however, evidence for this will require the analysis of other *Tenm4* alleles, which have abnormal body axis patterning or abnormal neural development [16].

## Conclusions

Our data show that *Tenm4* is required for mesoderm induction during gastrulation, and that gastrulation arrests at a stage prior to that in which Wnt signaling occurs. *Tenm4*^*m1/m1*^ mutants have a phenotype that is distinct from other mutants with gastrulation failure, but is most similar to that of β-catenin, which affects both the WNT and NODAL pathways. A GSK3β inhibitor rescued the ability of mutant embryos to form extraembryonic mesoderm and partially rescued the mutant embryos’ ability to form embryonic mesoderm. GSK3 can also affect adhesion complexes independent of WNT signaling. Given that *Tenm4* is a cell adhesion molecule that acts at neuronal synapses in flies, it is possible that cell-cell interactions are perturbed in the mutants, preventing the communication required for gastrulation to proceed. Further work will be required to fully understand the signaling pathways involved in this intriguing and important molecule.

## Methods

### Mouse strains and genotyping of adults and embryos

*l7Rn3*^*1777SB*^ and *l7Rn3*^*4323SB*^ (renamed *Tenm4*^*m1*^ and *Tenm4*^*m4*^, respectively) originated at the Oak Ridge National Laboratory and were obtained from Dr. E. M. Rinchik. The mice are maintained as heterozygous stocks by backcrossing with the inbred strain FRCH/Rl. The mutant alleles were induced on the BALB/cRl background, and are tightly linked to the *Tyr* (albino) locus. Embryos from timed matings were examined to determine the characteristics of mutants. Noon of the day of the appearance of the vaginal plug was designated E0.5. Embryos were examined visually and photographed, or fixed for histology or *in situ* hybridization. DNA from each embryo was extracted for genotyping.

TOPGAL mice were purchased from The Jackson Laboratory. The mice are maintained as heterozygous stocks by backcrossing with the inbred strain FVB. To obtain doubly heterozygous mice (*Tenm4*^m1^/+, *topgal*/+), were crossed with *Tenm4*^*m1*^ heterozygous mice and tails from all albino F1 mice were collected for x-gal staining. Furthermore, DNA from all x-gal positive tails (TOPGAL is expressed in the hair follicle) was extracted for genotyping.

Genotypes were determined by PCR analysis of genomic DNA from tail biopsies, embryonic yolk sac or whole embryos. Tails, yolk sacs or embryos were suspended in lysis buffer (50 mM Tris–HCl, pH 7.6, 20 mM NaCl, 0.1% SDS and 0.2 mg/ml proteinase K) and incubated at 55°C for 4–16 hours and 95°C for 10 minutes. For genotyping of sectioned samples from histological analysis, portions of sections were scratched and incubated with 50 μl of PCR reaction mix at 95°C for 10 min, then analyzed by PCR. A 122 bp fragment is amplified in BALB/cRl, 120 bp in FVB and a 108 bp in FRCH for D7Mit352 using the primers AGCCAATTGCAACCAAATTT (F) and AGCATGGAAAATTGACAATTCC (R). The PCR reaction was performed by denaturing at 94°C for 3 minutes, followed by 30 cycles of amplification: 94°C 1 minute, 55°C 30 seconds, 72°C 2 minutes and final extension at 72°C for 5 minutes.

### Histology and whole mount in situ hybridization

For histological examination, embryos were fixed in 4% paraformaldehyde overnight, embedded in paraffin wax, sectioned sagittally at 5–7 μm and stained with hematoxylin and eosin. For genotyping, parts of the sections were scratched prior to staining. Whole mount *in situ* hybridization using digoxigenin-labeled RNA probes was performed as described [61]. cDNA probes used for *in situ* hybridization were as described previously: *Brachyury*[23], *Foxa2*/*HNF3β*[26], *Hesx1*[62], *Lhx1*[27], *Otx2*[28], and *Bmp4*[29]. At least five mutant embryos were examined for each marker gene.

### BrdU labeling and TUNEL assays

BrdU (100 μg/gram of body weight) was injected intraperitoneally into pregnant females at E6.5. The females were sacrificed 20 min after injection and embryos were fixed with 4% paraformaldehyde, embedded in paraffin, and transversely sectioned at 5–7 μm. The sections were stained with mouse anti-BrdU antibody (Sigma), visualized by reaction with 3, 3^′^-diaminobenzidine and sections were counterstained with hematoxylin. For calculation of labeling index, we counted the BrdU positive cells in embryonic regions (5 slices from each embryo were used for average counting). TUNEL assays were performed with the In Situ Cell Death Detection Kit (Roche) and the sections were counterstained with methyl green.

### Embryonic teratoma assay

E6.5 embryos were isolated from matings of heterozygotes. After removal of the ectoplacental cone, embryos were transplanted into the testes of sibling males. One or seven week(s) later, the testes were removed and were fixed in 4% paraformaldehyde, and embedded in paraffin for histological analysis as described above. For genotyping of embryo-derived teratomas, parts of the teratoma were scratched from sectioned paraffin blocks.

### Whole embryo cultures

For whole embryo culture, embryos from matings between heterozygotes were dissected from decidual tissue, the parietal endoderm was removed and transferred to whole embryo culture medium containing (50% DMEM + 50% Rat serum obtained from Harlan) and placed into rolling culture. For the rescue experiment, E7.5 embryos from matings between heterozygotes were individually cultured as described above. 6-bromoindirubin-3^′^-oxime (BIO) and 1-methyl-6-bromoindirubin-3^′^-oxime (MeBIO) were purchased from Calbiochem/EMD Millipore (Billerica, MA). Some of cultured embryos were fixed with Carnoy’s fixative for histology, and the others were fixed with 2% glutaraldehyde for X-gal staining. For the ectoplacental cone (EPC) culture, E7.5 embryos were dissected from decidual tissues and EPCs were isolated and cultured on Petri dish in the medium containing high-glucose DMEM + 10% fetal bovine serum for 3 hours to remove adult blood cells from the EPCs. The EPC explants were transferred to collagen-coated dishes with fresh medium and were further cultured for two days.

### Blastocyst outgrowth cultures

Blastocysts (E3.5) were collected in M2 medium and then transferred to medium containing high-glucose DMEM, 20% fetal bovine serum, 0.1 mM β-mercaptoethanol, 1 mM sodium pyruvate, 1× non-essential amino acid, 2 mM glutamine, 100 units of penicillin per ml and 0.1 mg of streptomycin (ES medium lacking LIF) in 24-well plates coated with gelatin. The cultures were examined for attachment and growth, photographed and collected for genotyping.

### Immunohistochemistry

Pou5f1, Snai1 and E-cadherin antibodies were purchased from Santa Cruz Biotechnology, Inc. (Santa Cruz, CA) and N-cadherin antibody (MNCD2) was provided from the Developmental Studies Hybridoma Bank (University of Iowa, IA). Paraffin-embedded sections were cleared in histoclear and rehydrated through an ethanol series to PBS. Endogenous peroxidase activity was quenched by incubation of sections with 6% hydrogen peroxide in methanol for 1 hour at room temperature. For immunostaining of embryo outgrowth cultures, cultures were fixed in methanol at 4°C for 10 minutes, treated with 6% hydrogen peroxide in methanol and rehydrated through a methanol series to PBS. The sections or culture cells were then blocked with 10% normal sheep serum in PBS for an hour at room temperature and incubated with primary antibodies (diluted 1:500 – 1:2000) for an hour. After washing, the sections were incubated with horseradish-peroxidase-conjugated secondary antibodies diluted (1:200 – 1:1000) for an hour. The sections were then washed and reacted with peroxidase substrate.

For whole-mount immunostaining, embryos were fixed with 4% paraformaldehyde and transferred to methanol, treated with 6% hydrogen peroxide in methanol and rehydrated through a methanol series to PBS. Embryos were blocked with 10% normal sheep serum in PBS for 2 hours at room temperature and incubated with primary antibodies overnight at 4°C. After five washes with PBS for an hour each, embryos were incubated with horseradish-peroxidase-conjugated secondary antibodies overnight at 4°C, washed six times for an hour each and then placed in peroxidase substrate.

## Competing interests

The authors declare that they have no competing financial interests or non-competing personal interests in the publication of the data in this manuscript. These data have not been reported elsewhere.

## Authors’ contributions

HN carried out the experimental manipulations and wrote the initial draft of the paper. MJJ provided mutants, funding, edited and wrote the paper. RNC edited and wrote the paper. All authors read and approved the final manuscript.

## Supplementary Material

Additional file 1: Figure S1 Variation of *Brachyury *expression in *Tenm4*^*m1/m1 *^mutant embryos. No *Brachyury* expression was observed prior to E7.5 (see left panel). Some older *Tenm4*^*m1/m1 *^mutant embryos (E8.5) show slight *Brachyury *expression (Right panel), which might be background staining. Alternatively, some transcription may occur in the extraembryonic portion of embryos homozygous for the *Tenm4*^*m1 *^mutation. *Tenm4*^*m1/m1 *^mutant embryo arrests at the gastrulation stage and fail to develop a mesoderm. Histological paraffin section prepared from *Tenm4*^*m1/m1 *^mutant embryo, and stained with hematoxylin and eosin. Sagittal sections of *Tenm4*^*m1/m1 *^embryo at day E7.5. Thickness of section is 7 mm. Bar 100 μm. Click here for file

Additional file 2: Figure S2 Blastocyst outgrowth culture. E3.5 blastocysts collected from heterozygote matings were cultured on gelatin-coated dishes, examined for proliferation and genotyped after culture. No differences between the genotypes were observed before culture. Poor trophoblast growth and impaired proliferation of ICM were observed in *Tenm4*^*m1/m1 *^blastocyst culture. Photographs were taken at the first day of the culture (A, B), the third day (C, D) and the sixth day (E, F). A-C, wildtype blastocyst, D-F *Tenm4*^*m1/m1 *^mutant blastocyst. Click here for file
