# Supplementary figures and images for: Mouse Tenm4 is required for mesoderm induction
Source: BMC Dev Biol. 2013 Mar 25;13:9. doi: 10.1186/1471-213X-13-9 (PMC3614540; doi:10.1186/1471-213X-13-9)

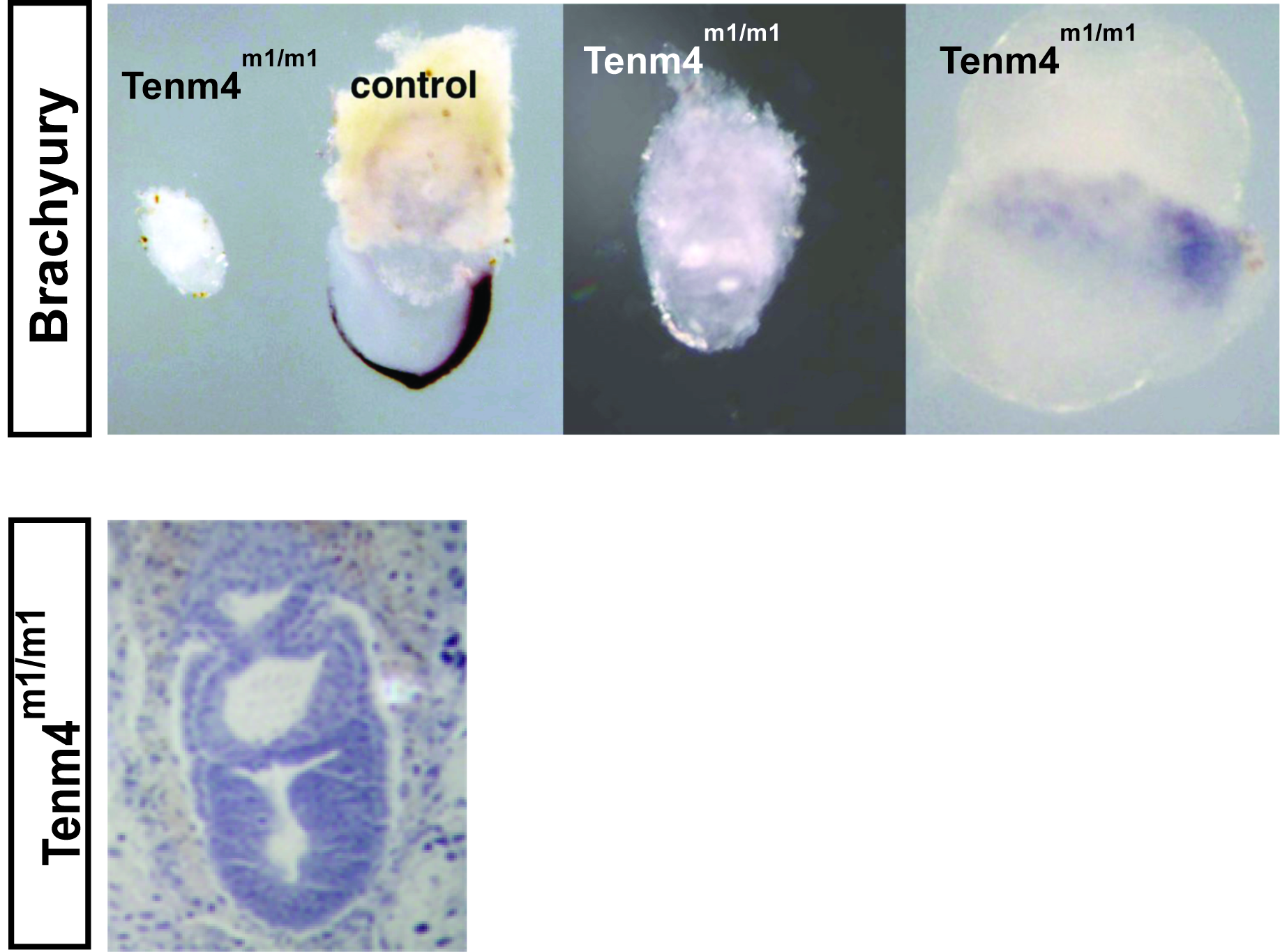

Supplement: Additional file 1: Figure S1 — Variation of Brachyury expression in Tenm4m1/m1 mutant embryos. No Brachyury expression was observed prior to E7.5 (see left panel). Some older Tenm4m1/m1 mutant embryos (E8.5) show slight Brachyury expression (Right panel), which might be background staining. Alternatively, some transcription may occur in the extraembryonic portion of embryos homozygous for the Tenm4m1 mutation. Tenm4m1/m1 mutant embryo arrests at the gastrulation stage and fail to develop a mesoderm. Histological paraffin section prepared from Tenm4m1/m1 mutant embryo, and stained with hematoxylin and eosin. Sagittal sections of Tenm4m1/m1 embryo at day E7.5. Thickness of section is 7 mm. Bar 100 μm. [file 1471-213X-13-9-S1.tiff]

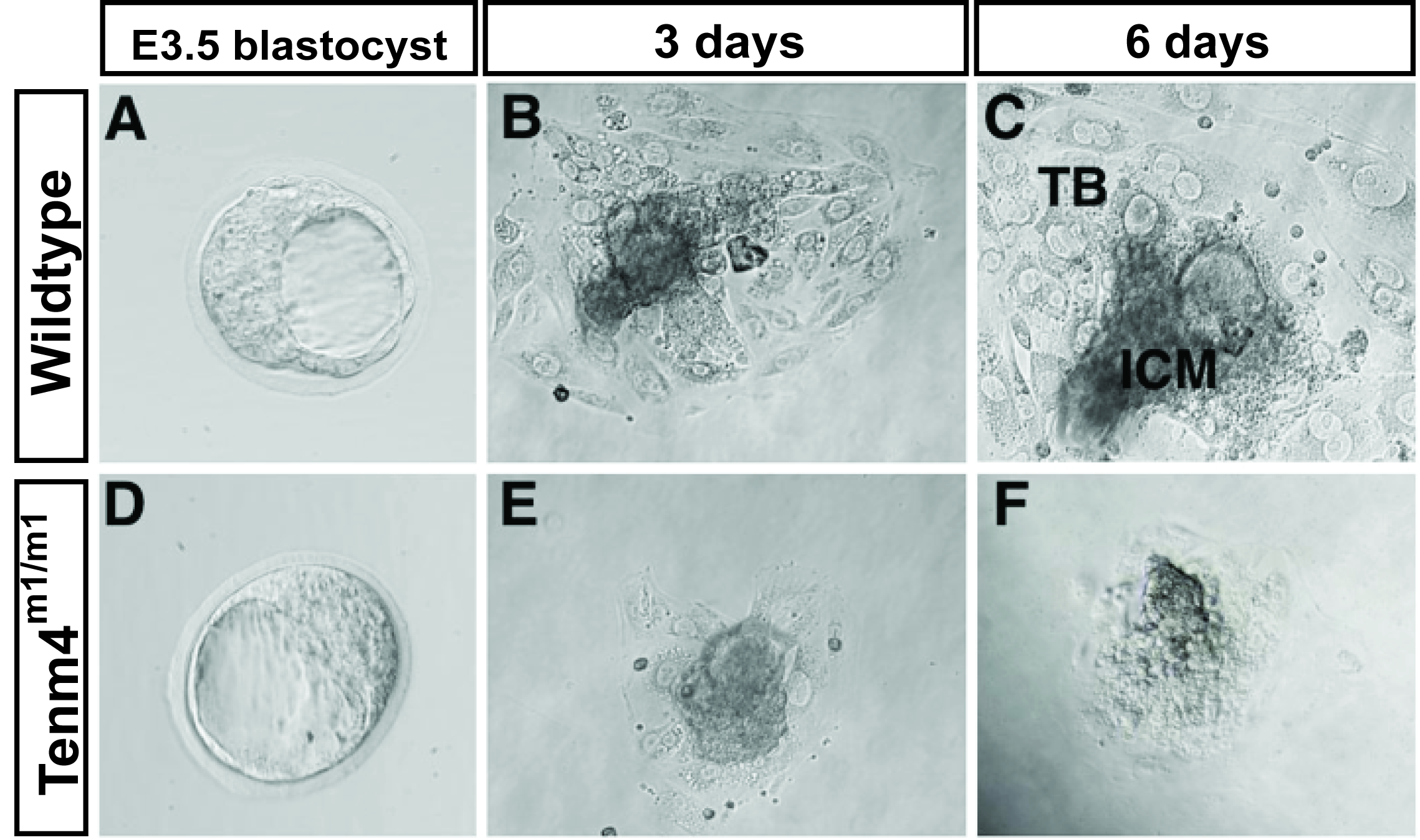

Supplement: Additional file 2: Figure S2 — Blastocyst outgrowth culture. E3.5 blastocysts collected from heterozygote matings were cultured on gelatin-coated dishes, examined for proliferation and genotyped after culture. No differences between the genotypes were observed before culture. Poor trophoblast growth and impaired proliferation of ICM were observed in Tenm4m1/m1 blastocyst culture. Photographs were taken at the first day of the culture (A, B), the third day (C, D) and the sixth day (E, F). A-C, wildtype blastocyst, D-F Tenm4m1/m1 mutant blastocyst. [file 1471-213X-13-9-S2.tiff]
